# Supplementary figures and images for: Morphological and olfactory tree traits influence the susceptibility and suitability of the apple species Malus domestica and M. sylvestris to the florivorous weevil Anthonomus pomorum (Coleoptera: Curculionidae)
Source: PeerJ. 2022 Jul 15;10:e13566. doi: 10.7717/peerj.13566 (PMC9291012; doi:10.7717/peerj.13566)

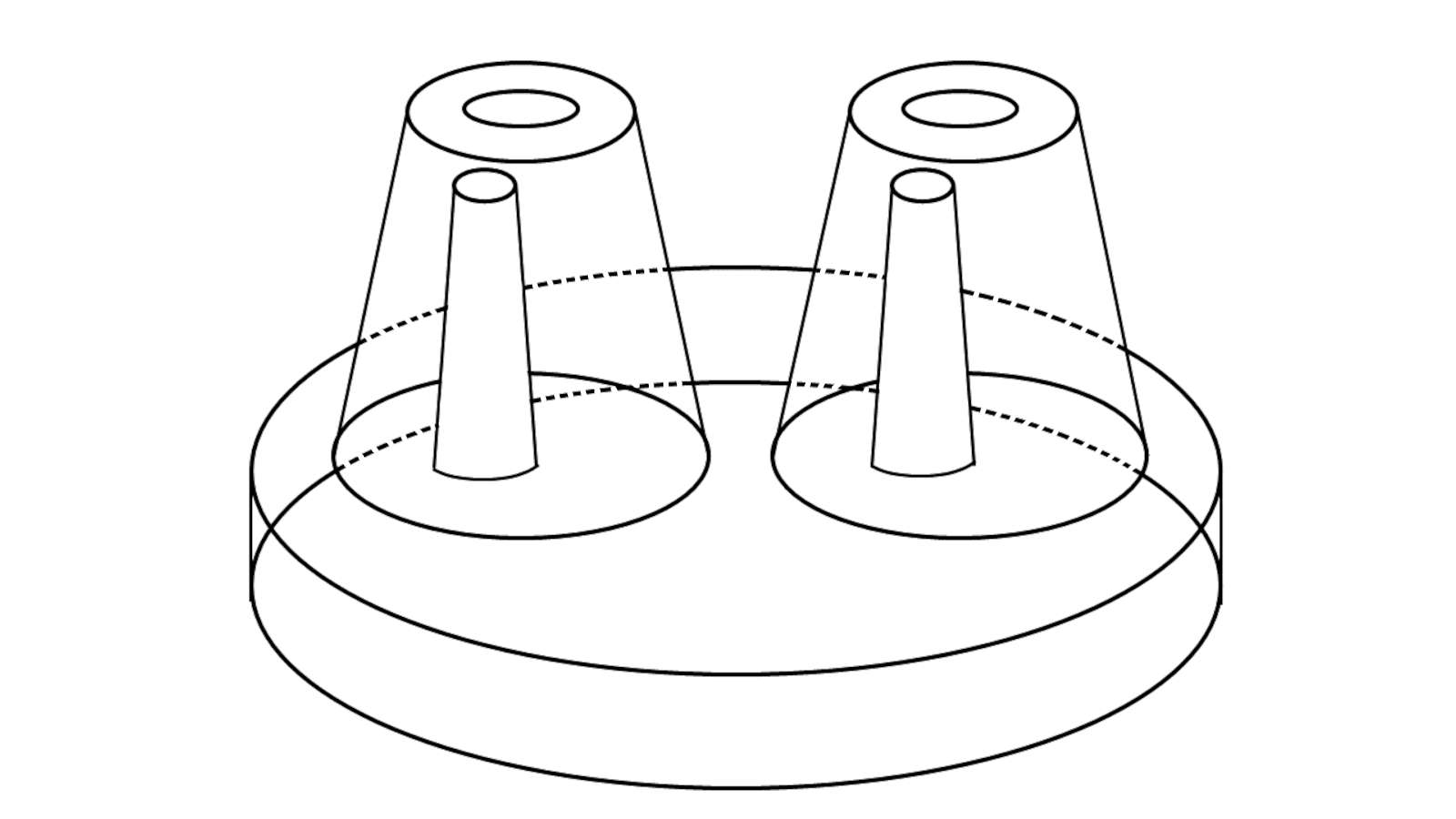

Supplement: Figure S1 [file peerj-10-13566-s001.png]

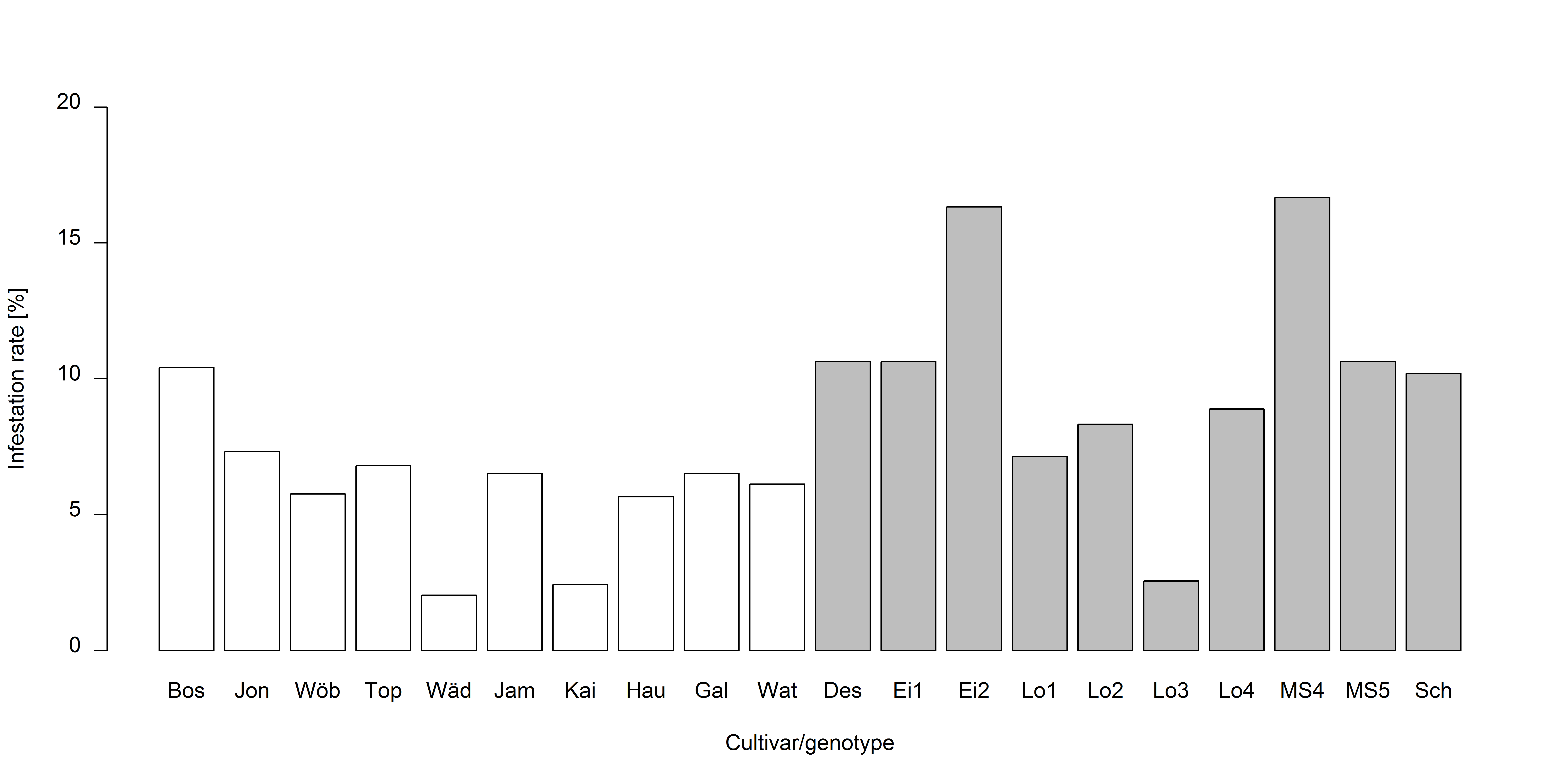

Supplement: Figure S2 — White columns: M. domestica, gray columns: M. sylvestris. One tree of each cultivar/genotype and ten blossom clusters per tree were examined (47.2 ± 3.94 (mean ± SD) blossom buds per tree). Bos = Roter Boskoop, Jon = Jonagold, Wöb = Wöbers Rambur, Top = Topaz, Wäd = Blauacher Wädenswil, Jam = James Grieve, Kai = Kaiser Wilhelm, Hau = Hauxapfel, Gal = Gala, Wat = Jonathan type “Watson”, Des = Destuben, Ei1 = Eimersmühle 1, Ei2 = Eimersmühle 2, Lo1 = Lochau 1, Lo2 = Lochau 2, Lo3 = Lochau 3, Lo4 = Lochau 4, MS4 = M.syl. 4, MS5 = M.syl. 5, Sch = Schlehenmühle. [file peerj-10-13566-s002.png]

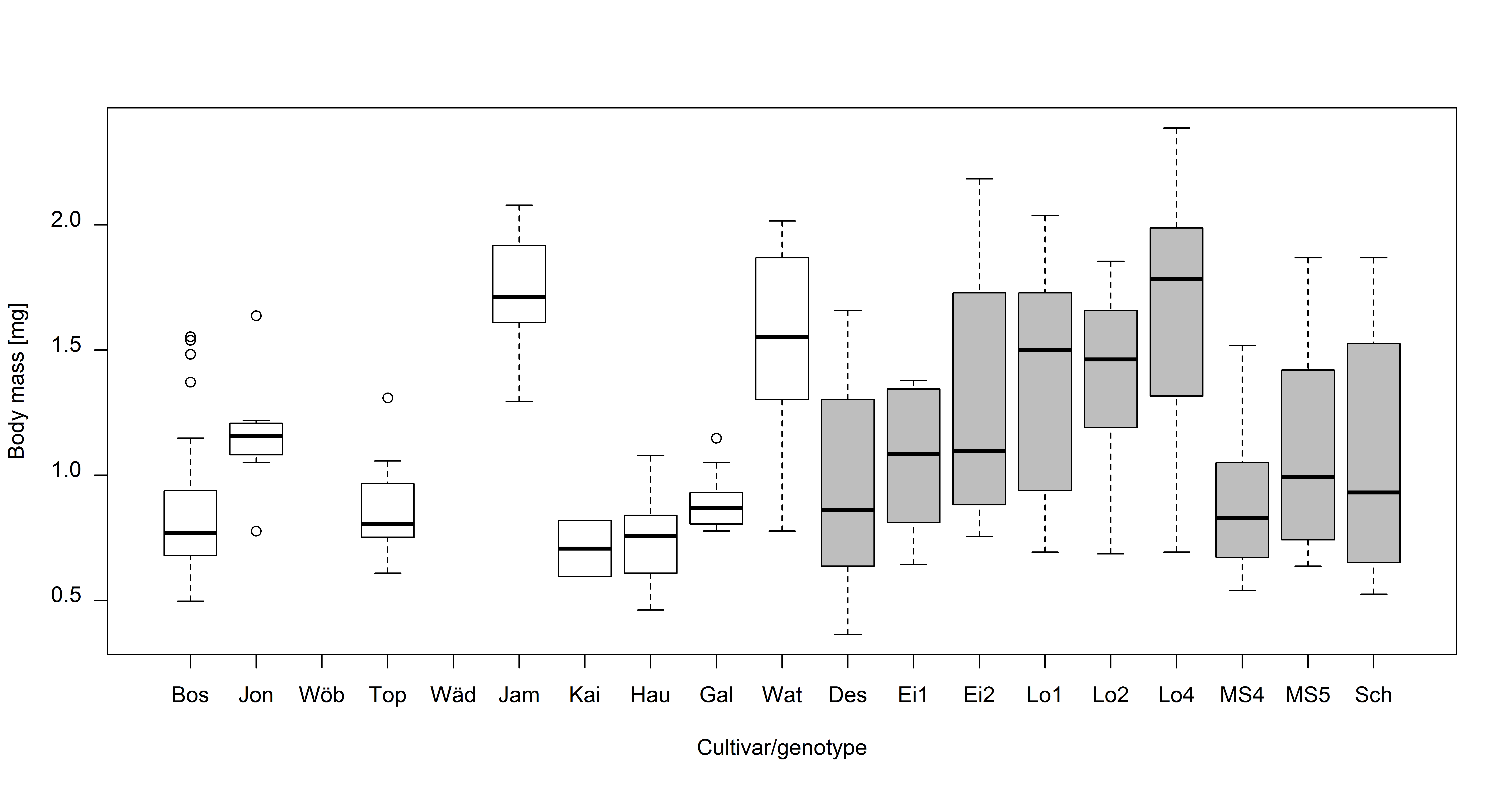

Supplement: Figure S3 — White boxes: M. domestica, gray boxes: M. sylvestris. 22.1 ± 14.06 (mean ± SD) weevils were examined per cultivar/genotype. Bos = Roter Boskoop, Jon = Jonagold, Wöb = Wöbers Rambur, Top = Topaz, Wäd = Blauacher Wädenswil, Jam = James Grieve, Kai = Kaiser Wilhelm, Hau = Hauxapfel, Gal = Gala, Wat = Jonathan type “Watson”, Des = Destuben, Ei1 = Eimersmühle 1, Ei2 = Eimersmühle 2, Lo1 = Lochau 1, Lo2 = Lochau 2, Lo4 = Lochau 4, MS4 = M.syl. 4, MS5 = M.syl. 5, Sch = Schlehenmühle. [file peerj-10-13566-s003.png]
