# Supplementary material for: Morphological and olfactory tree traits influence the susceptibility and suitability of the apple species Malus domestica and M. sylvestris to the florivorous weevil Anthonomus pomorum (Coleoptera: Curculionidae)
Source: PeerJ. 2022 Jul 15;10:e13566. doi: 10.7717/peerj.13566 (PMC9291012; doi:10.7717/peerj.13566)
Supplement: Table S1 — Diameter at breast height (DBH) refers to the diameter of the trunk measured at 130 cm above ground level. Canopy volume was estimated as a rotation ellipsoid with the height of the canopy as major axis and its diameter as minor axis (Hausmann, Samietz & Dorn, 2004a). [file peerj-10-13566-s004.docx]

| Cultivar/genotype | Species | Year of planting | DBH  [cm] | Tree height  [m] | Canopy volume  [m³] |
| --- | --- | --- | --- | --- | --- |
| Gala | *M. domestica* | 2002 | 12 | 6 | 30 |
| Wöbers Rambur | *M. domestica* | 2001 | 10 | 5 | 20 |
| Hauxapfel | *M. domestica* | 2002 | 15 | 7 | 70 |
| James Grieve | *M. domestica* | 1998 | 17 | 9 | 80 |
| Jonagold | *M. domestica* | 2001 | 8 | 4 | 16 |
| Jonathan type “Watson” | *M. domestica* | 2002 | 10 | 8 | 25 |
| Kaiser Wilhelm | *M. domestica* | 2002 | 12 | 6 | 40 |
| Roter Boskoop | *M. domestica* | 2001 | 15 | 8 | 60 |
| Blauacher Wädenswil | *M. domestica* | 2002 | 20 | 8 | 80 |
| Topaz | *M. domestica* | 2008 | 11 | 5 | 25 |
| M. syl. 4 | *M. sylvestris* | n. a. | 25 | 11 | 100 |
| M. syl. 5 | *M. sylvestris* | n. a. | 15 | 10 | 120 |
| Destuben | *M. sylvestris* | n. a. | 25 | 13 | 200 |
| Eimersmühle 1 | *M. sylvestris* | n. a. | 38 | 12 | 100 |
| Eimersmühle 2 | *M. sylvestris* | n. a. | 80 | 15 | 200 |
| Lochau 1 | *M. sylvestris* | n. a. | 10 | 7 | 25 |
| Lochau 2 | *M. sylvestris* | n. a. | 13 | 12 | 35 |
| Lochau 3 | *M. sylvestris* | n. a. | 18 | 12 | 60 |
| Lochau 4 | *M. sylvestris* | n. a. | 30 | 13 | 100 |
| Schlehenmühle | *M. sylvestris* | n. a. | 20 | 15 | 230 |
