# Supplementary material for: Morphological and olfactory tree traits influence the susceptibility and suitability of the apple species Malus domestica and M. sylvestris to the florivorous weevil Anthonomus pomorum (Coleoptera: Curculionidae)
Source: PeerJ. 2022 Jul 15;10:e13566. doi: 10.7717/peerj.13566 (PMC9291012; doi:10.7717/peerj.13566)
Supplement: Table S2 — Each individual tree was sampled three times on the same day without precipitation using headspace sorptive extraction (SBSE). Each sample was taken from a twig carrying 10 ± 1 blossom buds enclosed in a PET oven bag together with a 10-mm Gerstel©Twister. Sampling lasted for six hours. [file peerj-10-13566-s005.docx]

| Cultivar/genotype | Species | Plant accession | Year of grafting |
| --- | --- | --- | --- |
| Berlepsch | *M. domestica* | Berlepsch-3 | 2015 |
| Berlepsch | *M. domestica* | Berlepsch-10 | 2015 |
| Braeburn | *M. domestica* | Braeburn-9 | 2015 |
| Golden Delicious | *M. domestica* | GolDel-7 | 2015 |
| Golden Delicious | *M. domestica* | GolDel-4 | 2015 |
| Jakob Fischer | *M. domestica* | Fischer-12-u | 2015 |
| Jonagold | *M. domestica* | Jonagold-5 | 2015 |
| Jonagold | *M. domestica* | Jonagold-9 | 2015 |
| Topaz | *M. domestica* | Topaz-10 | 2015 |
| Topaz | *M. domestica* | Topaz-12 | 2015 |
| Eimersmühle 1 (E1) | *M. sylvestris* | E1-13 | 2015 |
| Eimersmühle 1 (E1) | *M. sylvestris* | E1-4 | 2015 |
| Lochau 1 (L1) | *M. sylvestris* | L1-11 | 2015 |
| Lochau 1 (L1) | *M. sylvestris* | L1-15 | 2016 |
| Neustaedtlein (N) | *M. sylvestris* | N-5 | 2015 |
| Neustaedtlein (N) | *M. sylvestris* | N-4 | 2016 |
| Schlehenmühle (RA) | *M. sylvestris* | S-7 | 2015 |
| Schlehenmühle (RA) | *M. sylvestris* | S-12 | 2015 |
