# Supplementary material for: Morphological and olfactory tree traits influence the susceptibility and suitability of the apple species Malus domestica and M. sylvestris to the florivorous weevil Anthonomus pomorum (Coleoptera: Curculionidae)
Source: PeerJ. 2022 Jul 15;10:e13566. doi: 10.7717/peerj.13566 (PMC9291012; doi:10.7717/peerj.13566)
Supplement: Table S3 [file peerj-10-13566-s006.docx]

| N | Components | GR^a^ | RI^b^ | *Malus sylvestris L Malus sylvestris E* | | | | | | *Malus domestica* | | | | |  |  |  |  |  |
| --- | --- | --- | --- | --- | --- | --- | --- | --- | --- | --- | --- | --- | --- | --- | --- | --- | --- | --- | --- |
|  |  |  |  | mean | SD mean | | | SD | | | mean | SD | |  |  |  |  |  |  |
| 1 | Pyridine | AR | <800 | 16,83 | 7,33 | 8,99 | | 8,18 | | | 13,43 | 12,09 | | | |  |  |  |  |
| 2 | Nonane | A | 900 | 4,38 | 1,36 | 1,74 | | 2,50 | | | 3,34 | 2,41 | | | |  |  |  |  |
| 3 | α-Pinene | MT | 931 | 1,19 | 1,16 | 0,61 | | 0,95 | | | 0,44 | 1,08 | | | |  |  |  |  |
| 4 | β-Myrcene | MT | 990 | 1,36a | 0,31 | 0,39b | | 0,53 | | | 0,28b | 0,69 | | | |  |  |  |  |
| 5 | Hexanoic acid | FA | 992 | 0,53 | 0,60 | 0,32 | | 0,55 | | | 0,39 | 0,72 | | | |  |  |  |  |
| 6 | U1 |  | 993 | 0,18 | 0,41 | 0,00 | |  | | | 0,06 | 0,15 | | | |  |  |  |  |
| 7 | U2 |  | 999 | 0,00 |  | 0,27 | | 0,46 | | | 0,37 | 0,58 | | | |  |  |  |  |
| 8 | α-Phellandrene | MT | 1000 | 0,00 |  | 0,09 | | 0,22 | | | 0,18 | 0,44 | | | |  |  |  |  |
| 9 | (Z)-3-Hexen-1-yl acetate | E | 1005 | 7,64a | 6,22 | 1,18b | | 2,09 | | | 2,82ab | 3,92 | | | |  |  |  |  |
| 10 | p-Cymene | ARMT | 1021 | 0,15 | 0,35 | 0,18 | | 0,29 | | | 0,16 | 0,40 | | | |  |  |  |  |
| 11 | D-Limonene | MT | 1024 | 0,15 | 0,34 | 0,20 | | 0,31 | | | 0,16 | 0,39 | | | |  |  |  |  |
| 12 | 2-Ethyl-1-hexanol | OH | 1028 | 0,40 | 0,37 | 0,54 | | 0,45 | | | 0,31 | 0,35 | | | |  |  |  |  |
| 13 | Propyl tiglate | E | 1034 | 1,72 | 1,07 | 0,90 | | 1,10 | | | 1,60 | 1,01 | | | |  |  |  |  |
| 14 | (E)- β-Ocimene | MT | 1047 | 0,69a | 0,80 | 0,00b | |  | | | 0,10ab | 0,24 | | | |  |  |  |  |
| 15 | γ-Terpinene | MT | 1056 | 0,00 |  | 0,01 | | 0,03 | | | 0,10 | 0,25 | | | |  |  |  |  |
| 16 | Acetophenone | AR | 1061 | 26,11 | 6,61 | 22,18 | | 10,66 | | | 30,46 | 12,21 | | | |  |  |  |  |
| 17 | Linalool | OMT | 1099 | 1,82b | 1,39 | 10,83a | | 5,35 | | | 2,40b | 2,27 | | | |  |  |  |  |
| 18 | Nonanal | Al | 1100 | 1,33 | 0,44 | 1,23 | | 1,37 | | | 1,34 | 0,61 | | | |  |  |  |  |
| 19 | 2-Phenylethanol | AR | 1110 | 0,54 | 0,74 | 0,16 | | 0,40 | | | 0,00 |  | | | |  |  |  |  |
| 20 | (E)-4,8-Dimethylnona-1,3,7-triene | HT | 1116 | 0,57 | 0,79 | 0,20 | | 0,23 | | | 0,18 | 0,45 | | | |  |  |  |  |
| 21 | Phenylacetonitrile | AR | 1134 | 0,00 |  | 0,00 | |  | | | 0,00 |  | | | |  |  |  |  |
| 22 | 3,3-Dimethylheptanoic acid | FA | 1148 | 0,24 | 0,33 | 0,35 | | 0,38 | | | 0,53 | 0,85 | | | |  |  |  |  |
| 23 | p-Cymen-8-ol | MT | 1182 | 0,61 | 0,56 | 0,08 | | 0,18 | | | 0,36 | 0,57 | | | |  |  |  |  |
| 24 | Methyl Salicylate | AR | 1190 | 0,49 | 1,10 | 0,56 | | 0,57 | | | 0,19 | 0,45 | | | |  |  |  |  |
| 25 | Hexyl butanoate | E | 1192 | 0,30 | 0,41 | 0,27 | | 0,32 | | | 0,11 | 0,26 | | | |  |  |  |  |
| 26 | Ethyl octanoate | E | 1197 | 0,00 |  | 0,17 | | 0,19 | | | 0,06 | 0,14 | | | |  |  |  |  |
| 27 | Dodecane | Al | 1200 | 0,00 |  | 0,23 | | 0,29 | | | 0,24 | 0,40 | | | |  |  |  |  |
| 28 | Decanal | Al | 1204 | 2,26 | 1,23 | 2,28 | | 2,91 | | | 2,49 | 1,17 | | | |  |  |  |  |
| 29 | β-Phenoxyethanol | OH | 1218 | 2,51 | 1,70 | 1,58 | | 2,18 | | | 2,34 | 1,69 | | | |  |  |  |  |
| 30 | Benzaldehyde | Al | 1248 | 0,81 | 0,29 | 0,52 | | 0,27 | | | 0,94 | 0,53 | | | |  |  |  |  |
| 31 | U3 |  | 1271 | 0,00 |  | 0,08 | | 0,20 | | | 0,00 |  | | | |  |  |  |  |
| 32 | U4 |  | 1276 | 0,00 |  | 0,13 | | 0,20 | | | 0,37 | 0,45 | | | |  |  |  |  |
| 33 | U5 |  | 1280 | 0,00 |  | 0,00 | |  | | | 0,00 |  | | | |  |  |  |  |
| 34 | Bornyl acetate | E | 1283 | 1,77 | 0,52 | 1,60 | | 0,96 | | | 2,32 | 0,92 | | | |  |  |  |  |
| 35 | Tridecane | A | 1300 | 0,11 | 0,25 | 0,00 | |  | | | 0,00 |  | | | |  |  |  |  |
| 36 | U7 |  | 1302 | 0,13 | 0,30 | 0,27 | | 0,53 | | | 0,21 | 0,33 | | | |  |  |  |  |
| 37 | U8 |  | 1305 | 0,00 |  | 0,00 | |  | | | 0,08 | 0,19 | | | |  |  |  |  |
| 38 | U9 |  | 1313 | 0,00 |  | 0,00 | |  | | | 0,06 | 0,15 | | | |  |  |  |  |
| 39 | U10 |  | 1319 | 0,00 |  | 0,00 | |  | | | 0,00 |  | | | |  |  |  |  |
| 40 | U11 |  | 1323 | 0,67 | 0,45 | 0,69 | | 0,46 | | | 0,90 | 0,62 | | | |  |  |  |  |
| 41 | U12 |  | 1327 | 0,00 |  | 0,32 | | 0,51 | | | 0,18 | 0,45 | | | |  |  |  |  |
| 42 | γ-Elemene | ST | 1335 | 0,00 |  | 0,00 | |  | | | 0,21 | 0,34 | | | |  |  |  |  |
| 43 | U13 |  | 1342 | 0,00 |  | 0,20 | | 0,24 | | | 0,04 | 0,10 | | | |  |  |  |  |
| 44 | U14 |  | 1343 | 0,00 |  | 0,05 | | 0,11 | | | 0,00 |  | | | |  |  |  |  |
| 45 | U15 |  | 1346 | 0,00 |  | 0,08 | | 0,20 | | | 0,08 | 0,20 | | | |  |  |  |  |
| 46 | U16 |  | 1354 | 4,59 | 1,83 | 3,26 | | 2,43 | | | 5,74 | 2,43 | | | |  |  |  |  |
| 47 | U17 |  | 1361 | 0,00 |  | 0,00 | |  | | | 0,09 | 0,23 | | | |  |  |  |  |
| 48 | U18 |  | 1364 | 0,00 |  | 1,38 | | 3,39 | | | 0,00 |  | | | |  |  |  |  |
| 49 | 3-Methyl-tridecane | A | 1370 | 0,00b |  | 0,49a | | 0,43 | | | 0,68ab | 1,29 | | | |  |  |  |  |
| 50 | ß-Bourbonene | ST | 1384 | 1,49a | 0,25 | 0,96b | | 0,53 | | | 1,71ab | 0,49 | | | |  |  |  |  |
| 51 | U19 |  | 1387 | 0,79 | 0,12 | 0,56 | | 0,32 | | | 0,71 | 0,41 | | | |  |  |  |  |
| 52 | U20 |  | 1394 | 1,46ab | 0,46 | 5,29a | | 2,80 | | | 1,15b | 1,64 | | | |  |  |  |  |
| 53 | Dodecanal | Al | 1407 | 0,00 |  | 0,00 | |  | | | 0,00 |  | | | |  |  |  |  |
| 54 | U21 |  | 1426 | 0,00 |  | 0,64 | | 0,52 | | | 0,47 | 0,82 | | | |  |  |  |  |
| 55 | ß-Copaene | ST | 1446 | 1,36 | 1,32 | 0,82 | | 0,99 | | | 1,66 | 1,46 | | | |  |  |  |  |
| 56 | U22 |  | 1448 | 0,29 | 0,40 | 0,56 | | 0,69 | | | 0,43 | 0,48 | | | |  |  |  |  |
|  |  |  |  |  | |  |  | |  | | | |  | | | |  |  |  |

^a^GR = group of chemical compound (A Alkane; AR aromatic compound; Al aldehyde; ARMT aromatic monoterpene; E ester; FA fatty acid conjugate; K ketone; MMT Monocyclic monoterpenoid; MT monoterpene; OH alcohol; OMT oxygenated monoterpene; ST sesquiterpenoid; HT homoterpene). Relative amounts of compounds with a different lettering (a,b) are significantly different from each other (P<0.05).

^b^ RI = retention index (DB5- fused silica capillary column 30 m× 0.25 mm i.d., 0.25 μm film thickness) experimentally determined using a homologue series of n-alkanes.

| N | Components | GR^a^ | RI^b^ | *Malus sylvestris L Malus sylvestris E* | | | | | | *Malus domestica* | | | | |  |  |  |  |  |
| --- | --- | --- | --- | --- | --- | --- | --- | --- | --- | --- | --- | --- | --- | --- | --- | --- | --- | --- | --- |
|  |  |  |  | mean | SD mean | | | SD | | | mean | | | SD | |  |  |  |  |
| 57 | Geranyl Acetone | K | 1450 | 1,17 | 2,63 | 4,01 | | 8,96 | | 0,66 | | 1,61 | | | |  |  |  |  |
| 58 | U23 |  | 1451 | 1,11 | 0,65 | 0,75 | | 0,52 | | 0,90 | | 0,19 | | | |  |  |  |  |
| 59 | p-Benzoquinone, 2,6-di-tert-butyl- | MMT | 1464 | 0,00 |  | 0,47 | | 1,15 | | 0,97 | | 2,38 | | | |  |  |  |  |
| 60 | U24 |  | 1474 | 0,00 |  | 0,77 | | 1,89 | | 0,86 | | 2,12 | | | |  |  |  |  |
| 61 | U25 |  | 1474 | 0,00 |  | 0,26 | | 0,31 | | 0,26 | | 0,45 | | | |  |  |  |  |
| 62 | γ-Muurolene | ST | 1475 | 1,40 | 1,35 | 1,81 | | 3,88 | | 1,21 | | 1,56 | | | |  |  |  |  |
| 63 | U26 |  | 1478 | 0,00b |  | 2,08a | | 1,42 | | 2,22ab | | 3,40 | | | |  |  |  |  |
| 64 | Germacrene D | ST | 1479 | 0,23 | 0,31 | 0,11 | | 0,28 | | 0,42 | | 0,34 | | | |  |  |  |  |
| 65 | 1-Pentadecene | AE | 1491 | 0,00b |  | 1,48a | | 1,18 | | 0,00b | |  | | | |  |  |  |  |
| 66 | Bicyclogermacrene | ST | 1495 | 1,00a | 0,23 | 0,19b | | 0,46 | | 0,93a | | 0,54 | | | |  |  |  |  |
| 67 | U27 |  | 1495 | 0,97 | 0,31 | 0,66 | | 0,46 | | 0,90 | | 0,27 | | | |  |  |  |  |
| 68 | Pentadecane | A | 1500 | 4,65 | 4,37 | 9,29 | | 9,36 | | 1,88 | | 1,23 | | | |  |  |  |  |
| 69 | α-Farnesene | ST | 1508 | 0,00 |  | 0,30 | | 0,33 | | 0,28 | | 0,49 | | | |  |  |  |  |
| 70 | γ-Cadinene | ST | 1512 | 0,00b |  | 0,55a | | 0,29 | | 0,29ab | | 0,51 | | | |  |  |  |  |
| 71 | δ-Cadinene | ST | 1523 | 0,38 | 0,37 | 0,41 | | 0,27 | | 0,64 | | 0,38 | | | |  |  |  |  |
| 72 | α-Cadinene | ST | 1536 | 1,15ab | 0,27 | 0,66b | | 0,46 | | 1,35a | | 0,38 | | | |  |  |  |  |
| 73 | U28 |  | 1541 | 0,00 |  | 0,06 | | 0,16 | | 0,11 | | 0,17 | | | |  |  |  |  |
| 74 | U29 |  | 1549 | 0,00 |  | 0,20 | | 0,32 | | 0,39 | | 0,35 | | | |  |  |  |  |
| 75 | U30 |  | 1552 | 0,00 | 2,63 | 0,11 | | 0,17 | | 0,35 | | 0,38 | | | |  |  |  |  |
|  |  |  |  |  | |  |  | |  | | | |  | | | |  |  |  |

^a^GR = group of chemical compound (A Alkane; AE Alkene; K ketone; MMT Monocyclic monoterpenoid; ST sesquiterpenoid). Relative amounts of compounds with a different lettering (a,b) are significantly different from each other (P<0.05).

^b^ RI = retention index (DB5- fused silica capillary column 30 m × 0.25 mm i.d., 0.25 μm film thickness) experimentally determined using a homologue series of n-alkanes
